# Private Key and Decoder Side Information for Secure and Private Source Coding †

**DOI:** 10.3390/e24121716

**Published:** 2022-11-24

**Authors:** Onur Günlü, Rafael F. Schaefer, Holger Boche, Harold Vincent Poor

**Affiliations:** 1 Information Coding Division, Linköping University, 58183 Linköping, Sweden; 2 Chair of Information Theory and Machine Learning, Technische Universität Dresden, 01062 Dresden, Germany; 3 BMBF Research Hub 6G-life, Technische Universität Dresden, 01062 Dresden, Germany; 4 Lehrstuhl für Theoretische Informationstechnik, TUM School of Computation, Information and Technology, Technical University of Munich, 80333 Munich, Germany; 5 CASA: Cyber Security in the Age of Large-Scale Adversaries Exzellenzcluster, Ruhr-Universität Bochum, 44780 Bochum, Germany; 6 BMBF Research Hub 6G-life, Technical University of Munich, 80333 Munich, Germany; 7 Munich Center for Quantum Science and Technology (MCQST), Schellingstr. 4, 80799 Munich, Germany; 8 Department of Electrical and Computer Engineering, Princeton University, Princeton, NJ 08544, USA

**Keywords:** information theoretic security, secure source coding, remote source, private key, side information

## Abstract

We extend the problem of secure source coding by considering a remote source whose noisy measurements are correlated random variables used for secure source reconstruction. The main additions to the problem are as follows: (1) all terminals noncausally observe a noisy measurement of the remote source; (2) a private key is available to all legitimate terminals; (3) the public communication link between the encoder and decoder is rate-limited; and (4) the secrecy leakage to the eavesdropper is measured with respect to the encoder input, whereas the privacy leakage is measured with respect to the remote source. Exact rate regions are characterized for a lossy source coding problem with a private key, remote source, and decoder side information under security, privacy, communication, and distortion constraints. By replacing the distortion constraint with a reliability constraint, we obtain the exact rate region for the lossless case as well. Furthermore, the lossy rate region for scalar discrete-time Gaussian sources and measurement channels is established. An achievable lossy rate region that can be numerically computed is also provided for binary-input multiple additive discrete-time Gaussian noise measurement channels.

## 1. Introduction

Consider multiple terminals that observe correlated random sequences and wish to reconstruct these sequences at another terminal, called a decoder, by sending messages through noiseless communication links, i.e., the distributed source coding problem [1]. A sensor network where each node observes a correlated random sequence that needs to be reconstructed at a distant node is a classic example of this problem [2] (p. 258). Similarly, function computation problems in which a fusion center observes messages sent by other nodes to compute a function are closely related problems that can be used to model various recent applications [3,4]. Since messages sent over communication links can be public, security constraints are imposed on these messages against an eavesdropper in the same network [5]. If all sent messages are available to the eavesdropper, it is necessary to provide an advantage to the decoder over the eavesdropper to enable secure source coding. Providing side information that is correlated with the sequences that should be reconstructed to the decoder can provide such an advantage over the eavesdropper that can also have side information, as in [6,7,8]. Allowing for the eavesdropper to access only a strict subset of all messages is also a method to enable secure distributed source coding, which was considered in [9,10,11]; see also [12], in which a similar method was applied to enable secure remote source reconstruction. Similarly, a private key that is shared by legitimate terminals and hidden from the eavesdropper can also provide such an advantage, as in [13,14].

Source coding models in the literature commonly assume that dependent multiletter random variables are available and should be compressed. For secret-key agreement [15,16] and secure function computation problems [17,18], which are instances of the source coding with the side information problem [19] (Section IV-B), the correlation between these multiletter random variables was posited in [20,21] to stem from an underlying ground truth that is a remote source, such that its noisy measurements are these dependent random variables. Such a remote source allows one to model the cause of correlation in a network, so we also posit that there is a remote source whose noisy measurements are used in the source coding problems discussed below, which is similar to the models in [22] (p. 78) and [23] (Figure 9). Furthermore, in the chief executive officer (CEO) problem [24], there is a remote source whose noisy measurements are encoded, such that a decoder can reconstruct the remote source by using encoder outputs. Our model is different from the model in the CEO problem, since in our model, the decoder aims to recover encoder observations rather than the remote source that is considered mainly to describe the cause of correlation between encoder observations. Thus, we define the *secrecy leakage* as the amount of information leaked to an eavesdropper about encoder observations. Since the remote source is common for all observations in the same network, we impose a *privacy leakage* constraint on the remote source because each encoder output observed by an eavesdropper leaks information about unused encoder observations, which might later cause secrecy leakage when the unused encoder observations are employed [25,26,27]; see [28,29,30] for joint secrecy and joint privacy constraints imposed due to multiple uses of the same source.

### 1.1. Summary of Contributions

We extend the lossless and lossy source coding rate region analyses by considering a remote source that should be kept private, decoder and eavesdropper side information, and a private key shared by the encoder and decoder. Considering that one encoder provides insights with enough richness to extend the results to multiple encoders [31], in this work, we consider the single encoder case. A summary of the main contributions is as follows.

- We characterize the lossy secure and private source coding region when noisy measurements of a remote source are observed by all terminals, and there is one private key available.
- Requiring reliable source reconstruction, we also characterize the rate region for the lossless secure and private source coding problem.
- A Gaussian remote source and independent additive Gaussian noise measurement channels are considered to establish their lossy rate region under squared error distortion.
- We provide an achievable lossy secure and private source coding region for a binary remote source and its measurements through additive Gaussian noise channels, which includes computable differential entropy terms.

### 1.2. Organization

This paper is organized as follows. In Section 2, we introduce the lossless and lossy secure and private source coding problems with decoder and eavesdropper side information and a private key under storage, secrecy, privacy, and reliability or distortion constraints. In Section 3, we characterize the rate regions for the introduced problems, which include three parts that correspond to different private key rate regimes. In Section 4, we evaluate the lossy rate region for Gaussian sources and channels with squared error distortion. In Section 5, we consider a binary modulated remote source measured through additive Gaussian noise channels and provide an inner bound for the lossy rate region with Hamming distortion. In Section 6, we provide the proof for the lossy secure and and private source coding region.

### 1.3. Notation

Uppercase *X* represents random variables and lowercase *x* their realizations from a set X, denoted by calligraphic letters. A discrete random variable *X* has probability distribution $P_{X}$ and a continuous random variable *X* probability density function (pdf) $p_{X}$. A subscript *i* denotes the position of a variable in a length-*n* sequence $X^{n}\;=\;X_{1},X_{2},\ldots ,X_{i},\ldots ,X_{n}$. Boldface uppercase $X={\left[X_{1},X_{2},\ldots \right]}^{T}$ represent vector random variables, where *T* denotes the transpose. [1:m] denotes the set {1,2,…,m} for an integer m≥1. Define ${\left[a\right]}^{-}=\min\left\{a,0\right\}$ for a∈R. Function $H_{b}\left(x\right)\;=\;-x\log x-\left(1\;-\;x\right)\log\left(1\;-\;x\right)$ is the binary entropy function, where logarithms are to the base 2. A binary symmetric channel (BSC) with crossover probability ϵ is denoted by BSC(ϵ). X∼Bern(β) with X={0,1} is a binary random variable with Pr[X=1]=β. The ∗-operator represents p∗q=(1−2q)p+q. Function Q(·) denotes the complementary cumulative distribution function of the standard Gaussian distribution. The function sgn(·) represents the signum function.

## 2. System Model

**Figure 1:**
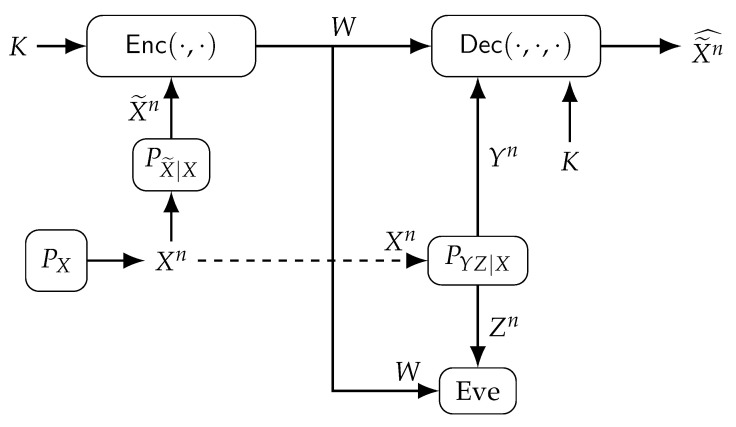
Source coding with noisy measurements $\left({\tilde{X}}^{n},Y^{n}\right)$ of a remote source $X^{n}$ and with a uniform private key *K* under privacy, secrecy, communication, and distortion constraints.

We consider the lossy source coding model with one encoder, one decoder, and an eavesdropper (Eve), depicted in Figure 1. The encoder Enc(·,·) observes a noisy measurement ${\tilde{X}}^{n}$ of an i.i.d. remote source $X^{n}∼P_{X}^{n}$ through a memoryless channel $P_{\tilde{X}|X}$ in addition to a private key $K\in [1:2^{nR_{0}}]$. The encoder output is an index *W* that is sent over a link with limited communication rate. Decoder Dec(·,·,·) observes index *W*, private key *K*, and another noisy measurement $Y^{n}$ of the same remote source $X^{n}$ through another memoryless channel $P_{YZ|X}$ in order to estimate ${\tilde{X}}^{n}$ as $\hat{{\tilde{X}}^{n}}$. The other noisy output $Z^{n}$ of $P_{YZ|X}$ is observed by Eve in addition to index *W*. Assume *K* is uniformly distributed, hidden from Eve, and independent of the source output and its noisy measurements. The source and measurement alphabets are finite sets.

We next define the rate region for the lossy secure and private source coding problem defined above.

**Definition** **1.** A *lossy* tuple $\left(R_{w},R_{s},R_{\ell },D\right)\;\in \;R_{\geq 0}^{4}$ is achievable given a private key with rate $R_{0}\;\geq \;0$, if for any δ>0 there exist n≥1, an encoder, and a decoder, such that

(1) $$\begin{array}{ccc} \frac{1}{n}\log|W|\leq R_{w}+\delta & \; & \left(\mathrm{storage}\right) \end{array}$$

(2) $$\begin{array}{ccc} \frac{1}{n}I\left({\tilde{X}}^{n};W|Z^{n}\right)\leq R_{s}+\delta & \; & \left(\sec\mathrm{recy}\right) \end{array}$$

(3) $$\begin{array}{ccc} \frac{1}{n}I\left(X^{n};W|Z^{n}\right)\leq R_{\ell }+\delta & \; & \left(\mathrm{privacy}\right) \end{array}$$

(4) $$\begin{array}{ccc} E\left[d\left({\tilde{X}}^{n},\hat{{\tilde{X}}^{n}}\left(Y^{n},W,K\right)\right)\right]\leq D+\delta & \; & \left(\mathrm{distortion}\right) \end{array}$$

where $d\left({\tilde{x}}^{n},\hat{{\tilde{x}}^{n}}\right)=\frac{1}{n}\sum_{i=1}^{n}d\left({\tilde{x}}_{i},\hat{{\tilde{x}}_{i}}\right)$ is a per-letter bounded distortion metric. The *lossy* secure and private source coding region $R_{D}$ is the closure of the set of all achievable lossy tuples. *◊*

In (2) and (3), we consider conditional mutual information terms to take account of unavoidable secrecy and privacy leakages due to Eve’s side information, i.e., $I({\tilde{X}}^{n};Z^{n})$ and $I(X^{n};Z^{n})$, respectively; see also [21,32]. Furthermore, we consider conditional mutual information terms rather than corresponding conditional entropy terms, the latter of which is used in [6,14,33,34,35], to characterize the secrecy and privacy leakages simplifies our analysis.

We next define the rate region for the lossless secure and private source coding problem.

**Definition** **2.** A *lossless* tuple $\left(R_{w},R_{s},R_{\ell }\right)\;\in \;R_{\geq 0}^{3}$ is achievable given a private key with rate $R_{0}\;\geq \;0$, if for any δ>0 there exist n≥1, an encoder, and a decoder, such that we have (1)–(3) and

(5) $$\begin{array}{ccc} \mathrm{Pr}\;\left[{\tilde{X}}^{n}\neq \hat{{\tilde{X}}^{n}}\left(Y^{n},W,K\right)\right]\;\leq \;\delta & \; & \;\;\;\;\;(\mathrm{reliability}). \end{array}$$

The *lossless* secure and private source coding region R is the closure of the set of all achievable lossless tuples. *◊*

## 3. Secure and Private Source Coding Regions

### 3.1. Lossy Source Coding

The lossy secure and and private source coding region $R_{D}$ is characterized below; see Section 6 for its proof.

Define ${\left[a\right]}^{-}=\min\left\{a,0\right\}$ for a∈R.

**Theorem** **1.** *For given $P_{X}$, $P_{\tilde{X}|X}$, $P_{YZ|X}$, and $R_{0}$, the region $R_{D}$ is the set of all rate tuples $\left(R_{w},R_{s},R_{\ell },D\right)$ satisfying*

(6) $$\begin{array}{cc} \; & R_{w}\geq I\left(U;\tilde{X}|Y\right) \end{array}$$

*and if $R_{0}<I\left(U;\tilde{X}|Y,V\right)$, then*

(7) $$\begin{array}{cc} \; & R_{s}\geq I\left(U;\tilde{X}|Z\right)+R^{\prime }-R_{0} \end{array}$$

(8) $$\begin{array}{cc} \; & R_{\ell }\geq I\left(U;X|Z\right)+R^{\prime }-R_{0} \end{array}$$

*where we have*

(9) $$\begin{array}{c} R^{\prime }={\left[I\left(U;Z|V,Q\right)-I\left(U;Y|V,Q\right)\right]}^{-} \end{array}$$

*and if $I\left(U;\tilde{X}|Y,V\right)\leq R_{0}<I\left(U;\tilde{X}|Y\right)$, then*

(10) $$\begin{array}{cc} \; & R_{s}\geq I\left(V;\tilde{X}|Z\right) \end{array}$$

(11) $$\begin{array}{cc} \; & R_{\ell }\geq I\left(V;X|Z\right) \end{array}$$

*and if $R_{0}\geq I\left(U;\tilde{X}|Y\right)$, then*

(12) $$\begin{array}{cc} \; & R_{s}\geq 0 \end{array}$$

(13) $$\begin{array}{cc} \; & R_{\ell }\geq 0 \end{array}$$

*for some*

(14) $$\begin{array}{c} P_{QVU\tilde{X}XYZ}=P_{Q|V}P_{V|U}P_{U|\tilde{X}}P_{\tilde{X}|X}P_{X}P_{YZ|X} \end{array}$$

*such that $E\left[d\left(\tilde{X},\hat{\tilde{X}}\left(U,Y\right)\right)\right]\leq D$ for some reconstruction function $\hat{\tilde{X}}\left(U,Y\right)$. The region $R_{D}$ is convexified by using the time-sharing random variable Q, required due to the ${\left[\cdot \right]}^{-}$ operation. One can limit the cardinalities to |Q| ≤ 2, $\left|V|\leq \right|\tilde{X}|+3$, and $\left|U\right|\leq \left(\right|\tilde{X}{\left|+3\right)}^{2}$.*

We remark that (12) and (13) show that one can simultaneously achieve *strong secrecy* and *strong privacy*, i.e., the conditional mutual information terms in (2) and (3), respectively, are negligible, by using a large private key *K*, which is a result missing in some recent works on secure source coding with a private key.

### 3.2. Lossless Source Coding

The lossless secure and and private source coding region R is characterized next; see below for a proof sketch.

**Proposition** **1.** *For given $P_{X}$, $P_{\tilde{X}|X}$, $P_{YZ|X}$, and $R_{0}$, the region R is the set of all rate tuples $\left(R_{w},R_{s},R_{\ell }\right)$ satisfying*

(15) $$\begin{array}{cc} \; & R_{w}\geq H\left(\tilde{X}|Y\right) \end{array}$$

*and if $R_{0}<H\left(\tilde{X}|Y,V\right)$, then*

(16) $$\begin{array}{cc} \; & R_{s}\geq H\left(\tilde{X}|Z\right)+R^{\prime \prime }-R_{0} \end{array}$$

(17) $$\begin{array}{cc} \; & R_{\ell }\geq I\left(\tilde{X};X|Z\right)+R^{\prime \prime }-R_{0} \end{array}$$

*where we have*

(18) $$\begin{array}{c} R^{\prime \prime }={\left[I\left(\tilde{X};Z|V,Q\right)-I\left(\tilde{X};Y|V,Q\right)\right]}^{-} \end{array}$$

*and if $H\left(\tilde{X}|Y,V\right)\leq R_{0}<H\left(\tilde{X}|Y\right)$, then*

(19) $$\begin{array}{cc} \; & R_{s}\geq I\left(V;\tilde{X}|Z\right) \end{array}$$

(20) $$\begin{array}{cc} \; & R_{\ell }\geq I\left(V;X|Z\right) \end{array}$$

*and if $R_{0}\geq H\left(\tilde{X}|Y\right)$, then*

(21) $$\begin{array}{cc} \; & R_{s}\geq 0 \end{array}$$

(22) $$\begin{array}{cc} \; & R_{\ell }\geq 0 \end{array}$$

*for some*

(23) $$\begin{array}{c} P_{QV\tilde{X}XYZ}=P_{Q|V}P_{V|\tilde{X}}P_{\tilde{X}|X}P_{X}P_{YZ|X}. \end{array}$$

*One can limit the cardinalities to |Q| ≤ 2 and $\left|V|\leq \right|\tilde{X}|+2$.*

**Proof** **Sketch.** The proof for the lossless region R follows from the proof for the lossy region $R_{D}$, given in Theorem 1 above, by choosing $U=\tilde{X}$, such that we have reconstruction function $\hat{\tilde{X}}\left(\tilde{X},Y\right)=\tilde{X}$, so we achieve D=0. Thus, the reliability constraint in (5) is satisfied because d(·,·) is a distortion metric. □

## 4. Gaussian Sources and Additive Gaussian Noise Channels

We evaluate the lossy rate region for a Gaussian example with squared error distortion by finding the optimal auxiliary random variable in the corresponding rate region. Consider a special lossy source coding case in which (i) there is no private key; (ii) the eavesdropper’s channel observation $Z^{n}$ is less noisy than the decoder’s channel observation $Y^{n}$, such that we obtain a lossy source coding region with a single auxiliary random variable that should be optimized.

We next define less noisy channels, considering $P_{YZ|X}$.

**Definition** **3** ([36]). *Z* (or eavesdropper) is *less noisy* than *Y* (or decoder) if

(24) $$\begin{array}{c} I(L;Z)\geq I(L;Y) \end{array}$$

holds for any random variable *L*, such that L−X−(Y,Z) form a Markov chain. *◊*

**Corollary** **1.** *For given $P_{X}$, $P_{\tilde{X}|X}$, $P_{YZ|X}$, and $R_{0}=0$, the region $R_{D}$ when the eavesdropper is less noisy than the decoder is the set of all rate tuples $\left(R_{w},R_{s},R_{\ell },D\right)$ satisfying*

(25) $$\begin{array}{cc} \; & R_{w}\geq I\left(U;\tilde{X}|Y\right)=I\left(U;\tilde{X}\right)-I\left(U;Y\right) \end{array}$$

(26) $$\begin{array}{cc} \; & R_{s}\geq I\left(U;\tilde{X}|Z\right)=I\left(U;\tilde{X}\right)-I\left(U;Z\right) \end{array}$$

(27) $$\begin{array}{cc} \; & R_{\ell }\geq I\left(U;X|Z\right)=I\left(U;X\right)-I\left(U;Z\right) \end{array}$$

*for some*

(28) $$\begin{array}{c} P_{U\tilde{X}XYZ}=P_{U|\tilde{X}}P_{\tilde{X}|X}P_{X}P_{YZ|X} \end{array}$$

*such that $E\left[d\left(\tilde{X},\hat{\tilde{X}}\left(U,Y\right)\right)\right]\;\;\leq \;\;D$ for some reconstruction function $\hat{\tilde{X}}\left(U,Y\right)$. One can limit the cardinality to $\left|U|\;\leq \;\right|\tilde{X}|\;+\;3$.*

**Proof** **Sketch.** The proof for Corollary 1 follows from the proof for Theorem 1 by considering the bounds in (6)–(8) since $R_{0}=0$. Furthermore, $R^{\prime }$ defined in (9) is 0 for the less noisy condition considered, which follows because (Q,V)−U−X−(Y,Z) form a Markov chain. □

Suppose the following scalar discrete-time Gaussian source and channel model for the lossy source coding problem depicted in Figure 1

(29) $$\begin{array}{cc} \; & X=\rho_{x}\tilde{X}+N_{x} \end{array}$$

(30) $$\begin{array}{cc} \; & Y=\rho_{y}X+N_{y} \end{array}$$

(31) $$\begin{array}{cc} \; & Z=\rho_{z}X+N_{z} \end{array}$$

where we have remote source X∼N(0,1), fixed correlation coefficients $\rho_{x},\rho_{y},\rho_{z}\in \left(-1,1\right)$, and additive Gaussian noise random variables

(32) $$\begin{array}{cc} \; & N_{x}\;∼\;N\left(0,1\;-\;\rho_{x}^{2}\right) \end{array}$$

(33) $$\begin{array}{cc} \; & N_{y}\;∼\;N\left(0,1\;-\;\rho_{y}^{2}\right) \end{array}$$

(34) $$\begin{array}{cc} \; & N_{z}\;∼\;N\left(0,1\;-\;\rho_{z}^{2}\right) \end{array}$$

such that $\left(\tilde{X},N_{x},N_{y},N_{z}\right)$ are mutually independent, and we consider the squared error distortion, i.e., $d\left(\tilde{x},\hat{\tilde{x}}\right)\;=\;{\left(\tilde{x}\;-\;\hat{\tilde{x}}\right)}^{2}$. Note that (29) is an inverse measurement channel $P_{X|\tilde{X}}$ that is a weighted sum of two independent Gaussian random variables, imposed to be able to apply the conditional entropy power inequality (EPI) [37] (Lemma II); see [20] (Theorem 3) and [38] (Section V) for binary symmetric inverse channel assumptions imposed to apply Mrs. Gerber’s lemma [39]. Suppose $|\rho_{z}\left|>\right|\rho_{y}|$, such that *Y* is less stochastically degraded than *Z*, since then there exists a random variable $\tilde{Y}$ such that $P_{\tilde{Y}|X}=P_{Y|X}$ and $P_{\tilde{Y}Z|X}\;=\;P_{Z|X}P_{\tilde{Y}|Z}$ [40] (Lemma 6), so *Z* is also less noisy than *Y* since less noisy channels constitute a strict superset of the set of stochastically-degraded channels and both channel sets consider only the conditional marginal probability distributions [2] (p. 121).

We next take the liberty to use the lossy rate region in Corollary 1, characterized for discrete memoryless channels, for the model in (29)–(31). This is common in the literature since there is a discretization procedure to extend the achievability proof to well-behaved continuous-alphabet random variables and the converse proof applies to arbitrary random variables; see [2] (Remark 3.8). For Gaussian sources and channels, we use differential entropy and eliminate the cardinality bound on the auxiliary random variable. The lossy source coding region for the model in (29)–(31) without a private key is given below.

**Proposition** **2.** *For the model in (29)–(31), such that $|\rho_{z}\left|>\right|\rho_{y}|$ and $R_{0}=0$, the region $R_{D}$ with squared error distortion is the set of all rate tuples $\left(R_{w},R_{s},R_{\ell },D\right)$ satisfying, for α∈(0,1],*

(35) $$\begin{array}{cc} \; & R_{w}\geq \frac{1}{2}\log\left(\frac{1-\rho_{x}^{2}\rho_{y}^{2}\left(1-\alpha \right)}{\alpha }\right) \end{array}$$

(36) $$\begin{array}{cc} \; & R_{s}\geq \frac{1}{2}\log\left(\frac{1-\rho_{x}^{2}\rho_{z}^{2}\left(1-\alpha \right)}{\alpha }\right) \end{array}$$

(37) $$\begin{array}{cc} \; & R_{\ell }\geq \frac{1}{2}\log\left(\frac{1-\rho_{x}^{2}\rho_{z}^{2}\left(1-\alpha \right)}{1-\rho_{x}^{2}\left(1-\alpha \right)}\right) \end{array}$$

(38) $$\begin{array}{cc} \; & D\geq \frac{\alpha (1-\rho_{x}^{2}\rho_{y}^{2})}{1-\rho_{x}^{2}\rho_{y}^{2}\left(1-\alpha \right)}. \end{array}$$

**Proof** **Sketch.** For the achievability proof, let U∼N(0,1−α) and Θ∼N(0,α), as in [41] ([Equation (32)]) and [42] (Appendix B), be independent random variables for some α∈(0,1] such that $\tilde{X}=U+\Theta$ and $U-\tilde{X}-X-\left(Y,Z\right)$ form a Markov chain. Choose the reconstruction function $\hat{\tilde{X}}\left(U,Y\right)$ as the minimum mean square error (MMSE) estimator, and given any fixed D>0, auxiliary random variables are chosen such that the distortion constraint is satisfied. We then have, for the squared error distortion,

(39) $$\begin{array}{c} D=E\left[{\left(\tilde{X}-\hat{\tilde{X}}\left(U,Y\right)\right)}^{2}\right]\overset{\left(a\right)}{=}\frac{1}{2\pi e}e^{2h(\tilde{X}|U,Y)} \end{array}$$

where equality in (a) is achieved because $\tilde{X}$ is Gaussian and the reconstruction function is the MMSE estimator [43] (Theorem 8.6.6). Define the covariance matrix of the vector random variable $\left[\tilde{X},U,Y\right]$ as $K_{\tilde{X}UY}$ and of [U,Y] as $K_{UY}$, respectively. We then have

(40) $$\begin{array}{cc} \; & h\left(\tilde{X}|U,Y\right)=h\left(\tilde{X},U,Y\right)-h\left(U,Y\right)\\ \; & =\frac{1}{2}\log\left(2\pi e\frac{\det(K_{\tilde{X}UY})}{\det(K_{UY})}\right) \end{array}$$

where det(·) is the determinant of a matrix; see also [12] (Section F). Combining (39) and (40), and calculating the determinants, we obtain

(41) $$\begin{array}{c} D=\frac{\alpha (1-\rho_{x}^{2}\rho_{y}^{2})}{1-\rho_{x}^{2}\rho_{y}^{2}\left(1-\alpha \right)}. \end{array}$$

One can also show that

(42) $$\begin{array}{cc} \; & I\left(U;\tilde{X}\right)\;=\;h\left(\tilde{X}\right)\;-\;h\left(\tilde{X}|U\right)\;=\;\frac{1}{2}\log\left(\frac{1}{\alpha }\right) \end{array}$$

(43) $$\begin{array}{cc} \; & I\left(U;X\right)\;=\;h\left(X\right)\;-\;h\left(X|U\right)\;=\;\frac{1}{2}\log\left(\frac{1}{1-\rho_{x}^{2}\left(1-\alpha \right)}\right) \end{array}$$

(44) $$\begin{array}{cc} \; & I\left(U;Y\right)\;=\;h\left(Y\right)\;-\;h\left(Y|U\right)\;=\;\frac{1}{2}\log\left(\frac{1}{1-\rho_{x}^{2}\rho_{y}^{2}\left(1-\alpha \right)}\right) \end{array}$$

(45) $$\begin{array}{cc} \; & I\left(U;Z\right)\;=\;h\left(Z\right)\;-\;h\left(Z|U\right)\;=\;\frac{1}{2}\log\left(\frac{1}{1-\rho_{x}^{2}\rho_{z}^{2}\left(1-\alpha \right)}\right). \end{array}$$

Thus, by calculating (25)–(27), the achievability proof follows.

For the converse proof, one can first show that

(46) $$\begin{array}{cc} \; & I\left(U;\tilde{X}\right)-I\left(U;Y\right)=h\left(Y|U\right)-h\left(\tilde{X}|U\right) \end{array}$$

(47) $$\begin{array}{cc} \; & I\left(U;\tilde{X}\right)-I\left(U;Z\right)=h\left(Z|U\right)-h\left(\tilde{X}|U\right) \end{array}$$

(48) $$\begin{array}{cc} \; & I(U;X)-I(U;Z)=h(Z|U)-h(X|U) \end{array}$$

which follow since $h\left(\tilde{X}\right)=h\left(X\right)=h\left(Y\right)=h\left(Z\right)$. Suppose

(49) $$\begin{array}{cc} \; & h\left(\tilde{X}|U\right)=\frac{1}{2}\log\left(2\pi e\alpha \right) \end{array}$$

for any α∈(0,1] that represents the unique variance of a Gaussian random variable; see [20] (Lemma 2) for a similar result applied to binary random variables. Thus, by applying the conditional EPI, we obtain

(50) $$\begin{array}{cc} \; & e^{2h(Y|U)}\overset{\left(a\right)}{=}e^{2h(\rho_{x}\rho_{y}\tilde{X}|U)}+e^{2h(\rho_{y}N_{x}+N_{y})}\\ \; & =2\pi e\left(\rho_{x}^{2}\rho_{y}^{2}\alpha +\rho_{y}^{2}\left(1-\rho_{x}^{2}\right)+1-\rho_{y}^{2}\right)\\ \; & =2\pi e\left(1-\rho_{x}^{2}\rho_{y}^{2}\left(1-\alpha \right)\right) \end{array}$$

where (a) follows because $U-\tilde{X}-\left(N_{x},N_{y}\right)$ form a Markov chain and $\left(N_{x},N_{y}\right)$ are independent of $\tilde{X}$, so $\left(N_{x},N_{y}\right)$ are independent of *U*, and equality is satisfied since, given *U*, $\rho_{x}\rho_{y}\tilde{X}$ and $\left(\rho_{y}N_{x}+N_{y}\right)$ are conditionally independent and they are Gaussian random variables, as imposed in (49) above; see [20] (Lemma 1 and Equation (28)) for a similar result applied to binary random variables by extending Mrs. Gerber’s lemma. Similarly, we have

(51) $$\begin{array}{cc} \; & e^{2h(Z|U)}=2\pi e\left(1-\rho_{x}^{2}\rho_{z}^{2}\left(1-\alpha \right)\right) \end{array}$$

which follows by replacing $\left(Y,\rho_{y},N_{y}\right)$ with $\left(Z,\rho_{z},N_{z}\right)$ in (50), respectively, because the channel $P_{Y|U}$ can be mapped to $P_{Z|U}$ with these changes due to (29)–(31) and the Markov chain relation $U-\tilde{X}-X-\left(Y,Z\right)$. Furthermore, we have

(52) $$\begin{array}{cc} \; & e^{2h(X|U)}\overset{\left(a\right)}{=}e^{2h(\rho_{x}\tilde{X}|U)}+e^{2h(N_{x})}\\ \; & =2\pi e\left(\rho_{x}^{2}\alpha +1-\rho_{x}^{2}\right)\\ \; & =2\pi e\left(1-\rho_{x}^{2}\left(1-\alpha \right)\right) \end{array}$$

where (a) follows because $N_{x}$ is independent of *U*, and equality is achieved since, given *U*, $\rho_{x}\tilde{X}$ and $N_{x}$ are conditionally independent and are Gaussian random variables. Therefore, by applying (46)–(52) to (25)–(27), the converse proof for (35)–(37) follows.

Next, consider

(53) $$\begin{array}{cc} \; & h\left(\tilde{X}|U,Y\right)=-I\left(U;\tilde{X}|Y\right)+h\left(\tilde{X}|Y\right)\\ \; & \overset{\left(a\right)}{=}-h\left(Y|U\right)+h\left(\tilde{X}|U\right)+h\left(Y|\tilde{X}\right)\\ \; & \overset{\left(b\right)}{=}\frac{1}{2}\log\left(\frac{\alpha }{1-\rho_{x}^{2}\rho_{y}^{2}\left(1-\alpha \right)}\right)+h\left(\rho_{x}\rho_{y}\tilde{X}\;+\;\rho_{y}N_{x}\;+\;N_{y}|\tilde{X}\right)\\ \; & \overset{\left(c\right)}{=}\frac{1}{2}\log\left(\frac{\alpha }{1-\rho_{x}^{2}\rho_{y}^{2}\left(1-\alpha \right)}\right)+h\left(\rho_{y}N_{x}\;+\;N_{y}\right)\\ \; & =\frac{1}{2}\log\left(2\pi e\frac{\alpha (\rho_{y}^{2}\left(1-\rho_{x}^{2}\right)+\left(1-\rho_{y}^{2}\right))}{1-\rho_{x}^{2}\rho_{y}^{2}\left(1-\alpha \right)}\right)\\ \; & =\frac{1}{2}\log\left(2\pi e\frac{\alpha (1-\rho_{x}^{2}\rho_{y}^{2})}{1-\rho_{x}^{2}\rho_{y}^{2}\left(1-\alpha \right)}\right) \end{array}$$

where (a) follows by (25) and (46), and since $h\left(Y\right)=h\left(\tilde{X}\right)$, (b) follows by (49) and (50), and (c) follows because $\left(N_{x},N_{y}\right)$ are independent of $\tilde{X}$. Furthermore, for any random variable $\tilde{X}$ and reconstruction function $\hat{\tilde{X}}\left(U,Y\right)$, we have [43] (Theorem 8.6.6)

(54) $$\begin{array}{c} E\left[{\left(\tilde{X}-\hat{\tilde{X}}\left(U,Y\right)\right)}^{2}\right]\geq \frac{1}{2\pi e}e^{2h(\tilde{X}|U,Y)}. \end{array}$$

Combining the distortion constraint given in Corollary 1 with (53) and (54), the converse proof for (38) follows. □

## 5. Multiple Binary-input Additive Gaussian Noise Channels

Consider next a binary remote source X∈{−1,1} and its binary noisy measurement $\tilde{X}\in \left\{-1,1\right\}$ observed by the encoder, which represents a practical setting with binary quantizations. For instance, a static random-access memory (SRAM) start-up output at a nominal temperature is a binary value obtained by quantizing sums of Gaussian random variables [28,44]. Suppose the noisy channel $P_{YZ|X}$ outputs consist of a single discrete-time additive Gaussian noise channel output *Y* observed by the decoder and two independent discrete-time additive Gaussian noise channel outputs $Z={\left[Z_{1},Z_{2}\right]}^{T}$ observed by the eavesdropper, in which the eavesdropper obtains more information by measuring the remote source twice. Furthermore, assume that *X* is uniformly distributed, the binary channel $P_{\tilde{X}|X}$ is symmetric such that $\mathrm{Pr}[\tilde{X}\neq X]=p$ for p∈[0,1], and we also have

(55) $$\begin{array}{cc} Y= & \rho_{y}X+N_{y} \end{array}$$

(56) $$\begin{array}{cc} Z= & \left[\begin{array}{c} Z_{1}\\ Z_{2} \end{array}\right]=\rho_{z}X\left[\begin{array}{c} 1\\ 1 \end{array}\right]+\left[\begin{array}{c} N_{z_{1}}\\ N_{z_{2}} \end{array}\right] \end{array}$$

where we have fixed correlation coefficients $\rho_{y},\rho_{z}\in \left(-1,1\right)$ and additive Gaussian noise random variables

(57) $$\begin{array}{cc} \; & N_{y}\;∼\;N\left(0,1\;-\;\rho_{y}^{2}\right) \end{array}$$

(58) $$\begin{array}{cc} \; & N_{z_{1}}\;∼\;N\left(0,1\;-\;\rho_{z}^{2}\right) \end{array}$$

(59) $$\begin{array}{cc} \; & N_{z_{2}}\;∼\;N\left(0,1\;-\;\rho_{z}^{2}\right) \end{array}$$

such that $\left(X,N_{y},N_{z_{1}},N_{z_{2}}\right)$ are mutually independent. Consider the Hamming distortion, i.e., $d\left(\tilde{x},\hat{\tilde{x}}\right)\;=\;1\left\{\tilde{x}\neq \hat{\tilde{x}}\right\}$. Impose the condition $|\rho_{z}\left|>\right|\rho_{y}|$ such that $Z_{1}$ and $Z_{2}$ are less noisy than *Y*, so Z is also less noisy than *Y*, which follows by applying similar steps as being applied in Section 4. Thus, for $R_{0}=0$, the region $R_{D}$ characterized in Corollary 1 is also valid for such binary-input additive Gaussian noise channels when one replaces *Z* with Z. A computable achievable lossy secure and private source coding region for such channels is given next.

**Proposition** **3.** *For the setting with multiple binary-input additive Gaussian noise channels, defined above, such that $|\rho_{z}\left|>\right|\rho_{y}|$ and $R_{0}=0$, the region $R_{D}$ with Hamming distortion includes the set of all rate tuples $\left(R_{w},R_{s},R_{\ell },D\right)$ satisfying, for an independent random variable C∼Bern(p∗q) with any q∈[0,0.5] and for any λ∈[0,1],*

(60) $$\begin{array}{c} R_{w}\geq \lambda \left(1-H_{b}\left(q\right)-h\left(\rho_{y}X+N_{y}\right)+h\left(\rho_{y}\left(1\;-\;2C\right)+N_{y}\right)\right) \end{array}$$

(61) $$\begin{array}{l} R_{s}\geq \lambda \left(1-H_{b}\left(q\right)-h\left(\left[\begin{array}{c} \rho_{z}X+N_{z_{1}}\\ \rho_{z}X+N_{z_{2}} \end{array}\right]\right)+h\left(\left[\begin{array}{c} \rho_{z}\left(1\;-\;2C\right)+N_{z_{1}}\\ \rho_{z}\left(1\;-\;2C\right)+N_{z_{2}} \end{array}\right]\right)\right) \end{array}$$

(62) $$\begin{array}{l} R_{\ell }\geq \lambda \left(1-H_{b}\left(p*q\right)-h\left(\left[\begin{array}{c} \rho_{z}X+N_{z_{1}}\\ \rho_{z}X+N_{z_{2}} \end{array}\right]\right)+h\left(\left[\begin{array}{c} \rho_{z}\left(1\;-\;2C\right)+N_{z_{1}}\\ \rho_{z}\left(1\;-\;2C\right)+N_{z_{2}} \end{array}\right]\right)\right) \end{array}$$

(63) $$\begin{array}{c} D\geq \lambda q+\left(1-\lambda \right)\left(p*Q\left(\frac{\rho_{y}}{\sqrt{1-\rho_{y}^{2}}}\right)\right) \end{array}$$

*where random variable $Y=\left(\rho_{y}X+N_{y}\right)$ has pdf*

(64) $$\begin{array}{c} \frac{1}{2}\frac{\left(e^{-\frac{{\left(y+\rho_{y}\right)}^{2}}{2(1-\rho_{y}^{2})}}+e^{-\frac{{\left(y-\rho_{y}\right)}^{2}}{2(1-\rho_{y}^{2})}}\right)}{\sqrt{2\pi (1-\rho_{y}^{2})}} \end{array}$$

*the random variable $\bar{Y}=\left(\rho_{y}\left(1\;-\;2C\right)+N_{y}\right)$ has pdf*

(65) $$\begin{array}{c} \left(p*q\right)\frac{e^{-\frac{{\left(\bar{y}+\rho_{y}\right)}^{2}}{2(1-\rho_{y}^{2})}}}{\sqrt{2\pi (1-\rho_{y}^{2})}}+\left(1\;-\;\left(p*q\right)\right)\frac{e^{-\frac{{\left(\bar{y}-\rho_{y}\right)}^{2}}{2(1-\rho_{y}^{2})}}}{\sqrt{2\pi (1-\rho_{y}^{2})}} \end{array}$$

*the vector random variable $\left[\begin{array}{c} Z_{1}\\ Z_{2} \end{array}\right]=\left(\left[\begin{array}{c} \rho_{z}X+N_{z_{1}}\\ \rho_{z}X+N_{z_{2}} \end{array}\right]\right)$ has joint pdf*

(66) $$\begin{array}{c} \frac{1}{2}\frac{\left(e^{-\frac{\left({\left(z_{1}+\rho_{z}\right)}^{2}+{\left(z_{2}+\rho_{z}\right)}^{2}\right)}{2(1-\rho_{z}^{2})}}+e^{-\frac{\left({\left(z_{1}-\rho_{z}\right)}^{2}+{\left(z_{2}-\rho_{z}\right)}^{2}\right)}{2(1-\rho_{z}^{2})}}\right)}{2\pi (1-\rho_{z}^{2})} \end{array}$$

*and the vector random variable $\left[\begin{array}{c} {\bar{Z}}_{1}\\ {\bar{Z}}_{1} \end{array}\right]=\left(\left[\begin{array}{c} \rho_{z}\left(1\;-\;2C\right)+N_{z_{1}}\\ \rho_{z}\left(1\;-\;2C\right)+N_{z_{2}} \end{array}\right]\right)$ has joint pdf*

(67) $$\begin{array}{c} \left(p*q\right)\frac{e^{-\frac{\left({\left({\bar{z}}_{1}+\rho_{z}\right)}^{2}+{\left({\bar{z}}_{2}+\rho_{z}\right)}^{2}\right)}{2(1-\rho_{z}^{2})}}}{2\pi (1-\rho_{z}^{2})}+\left(1\;-\;\left(p*q\right)\right)\frac{e^{-\frac{\left({\left({\bar{z}}_{1}-\rho_{z}\right)}^{2}+{\left({\bar{z}}_{2}-\rho_{z}\right)}^{2}\right)}{2(1-\rho_{z}^{2})}}}{2\pi (1-\rho_{z}^{2})}. \end{array}$$

**Proof.**  We first evaluate (25)–(27) by choosing a binary uniformly distributed *U* and a channel $P_{\tilde{X}|U}$ such that $\mathrm{Pr}[\tilde{X}\neq U]=q$ for any q∈[0,0.5]. We have

(68) $$\begin{array}{cc} \; & I\left(U;\tilde{X}\right)=H\left(\tilde{X}\right)-H\left(\tilde{X}|U\right)\overset{\left(a\right)}{=}1-H_{b}\left(q\right) \end{array}$$

(69) $$\begin{array}{cc} \; & I\left(U;X\right)=H\left(X\right)-H\left(X|U\right)\overset{\left(b\right)}{=}1-H_{b}\left(p*q\right) \end{array}$$

where (a) and (b) follow by relabeling the input and output symbols to represent the channels $P_{\tilde{X}|U}$ and $P_{X|\tilde{X}}$ as BSC(q) and BSC(p), respectively, which follows since entropy is preserved under a bijective mapping for discrete random variables. For relabeled symbols, the channel $P_{X|U}$ is a BSC(p∗q) since it is a concatenation of two BSCs, so denote the independent random noise component in this channel as C∼Bern(p∗q). Then, we obtain

(70) $$\begin{array}{c} h\left(Y|U\right)=h\left(\rho_{y}X+N_{y}|U\right)\overset{\left(a\right)}{=}h\left(\rho_{y}\left(1\;-\;2C\right)+N_{y}\right)=h\left(\bar{Y}\right) \end{array}$$

where (a) follows since symbols {−1,1} correspond to the antipodal modulation of binary symbols, and since $\left(C,N_{y},U\right)$ are mutually independent. One can compute (70) numerically by using the pdf

(71) $$\begin{array}{c} p_{\bar{Y}}\left(\bar{y}\right)=\sum_{c=0}^{1}P_{C}\left(c\right)p_{\bar{Y}|C}\left(\bar{y}|c\right)=\left(p*q\right)\frac{e^{-\frac{{\left(\bar{y}+\rho_{y}\right)}^{2}}{2(1-\rho_{y}^{2})}}}{\sqrt{2\pi (1-\rho_{y}^{2})}}+\left(1\;-\;\left(p*q\right)\right)\frac{e^{-\frac{{\left(\bar{y}-\rho_{y}\right)}^{2}}{2(1-\rho_{y}^{2})}}}{\sqrt{2\pi (1-\rho_{y}^{2})}}. \end{array}$$

Similarly, we can compute

(72) $$\begin{array}{c} h\left(Y\right)=h\left(\rho_{y}X+N_{y}\right) \end{array}$$

numerically by using the pdf

(73) $$\begin{array}{c} p_{Y}\left(y\right)=\sum_{x\in \{-1,1\}}P_{X}\left(x\right)p_{Y|X}\left(y|x\right)=\frac{1}{2}\frac{\left(e^{-\frac{{\left(y+\rho_{y}\right)}^{2}}{2(1-\rho_{y}^{2})}}+e^{-\frac{{\left(y-\rho_{y}\right)}^{2}}{2(1-\rho_{y}^{2})}}\right)}{\sqrt{2\pi (1-\rho_{y}^{2})}}. \end{array}$$

Next, consider

(74) $$\begin{array}{c} h\left(Z|U\right)=h\left(\left(\rho_{z}X\left[\begin{array}{c} 1\\ 1 \end{array}\right]+\left[\begin{array}{c} N_{z_{1}}\\ N_{z_{2}} \end{array}\right]\right)|U\right)\overset{\left(a\right)}{=}h\left(\left[\begin{array}{c} \rho_{z}\left(1\;-\;2C\right)+N_{z_{1}}\\ \rho_{z}\left(1\;-\;2C\right)+N_{z_{2}} \end{array}\right]\right)=h\left(\left[\begin{array}{c} {\bar{Z}}_{1}\\ {\bar{Z}}_{2} \end{array}\right]\right) \end{array}$$

where (a) follows since $\left(C,N_{z_{1}},N_{z_{2}},U\right)$ are mutually independent. Denote

(75) $$\begin{array}{cc} \; & \bar{Z}={\left[{\bar{Z}}_{1},{\bar{Z}}_{2}\right]}^{T}. \end{array}$$

We can compute (74) numerically by using the joint pdf

(76) $$\begin{array}{cc} \; & p_{\bar{Z}}\left(\bar{z}\right)=p_{{\bar{Z}}_{1}{\bar{Z}}_{2}}\left({\bar{z}}_{1},{\bar{z}}_{2}\right)=\sum_{c=0}^{1}P_{C}\left(c\right)p_{{\bar{Z}}_{1}{\bar{Z}}_{2}|C}\left({\bar{z}}_{1},{\bar{z}}_{2}|c\right)\\ \; & =\left(p*q\right)\frac{e^{-\frac{\left({\left({\bar{z}}_{1}+\rho_{z}\right)}^{2}+{\left({\bar{z}}_{2}+\rho_{z}\right)}^{2}\right)}{2(1-\rho_{z}^{2})}}}{2\pi (1-\rho_{z}^{2})}+\left(1\;-\;\left(p*q\right)\right)\frac{e^{-\frac{\left({\left({\bar{z}}_{1}-\rho_{z}\right)}^{2}+{\left({\bar{z}}_{2}-\rho_{z}\right)}^{2}\right)}{2(1-\rho_{z}^{2})}}}{2\pi (1-\rho_{z}^{2})} \end{array}$$

which follows since $\bar{Z}|C$ is a jointly Gaussian vector random variable with independent components ${\bar{Z}}_{1}|C$ and ${\bar{Z}}_{2}|C$, since every scalar linear combination of the components is Gaussian; see [45] (Theorem 1). Similarly, we can compute

(77) $$\begin{array}{c} h\left(Z\right)=h\left(\left[\begin{array}{c} \rho_{z}X+N_{z_{1}}\\ \rho_{z}X+N_{z_{2}} \end{array}\right]\right) \end{array}$$

numerically by using the joint pdf

(78) $$\begin{array}{cc} \; & p_{Z}\left(z\right)=p_{Z_{1}Z_{2}}\left(z_{1},z_{2}\right)=\sum_{x\in \{-1,1\}}P_{X}\left(x\right)p_{Z_{1}Z_{2}|X}\left(z_{1},z_{2}|x\right)\\ \; & =\frac{1}{2}\frac{\left(e^{-\frac{\left({\left(z_{1}+\rho_{z}\right)}^{2}+{\left(z_{2}+\rho_{z}\right)}^{2}\right)}{2(1-\rho_{z}^{2})}}+e^{-\frac{\left({\left(z_{1}-\rho_{z}\right)}^{2}+{\left(z_{2}-\rho_{z}\right)}^{2}\right)}{2(1-\rho_{z}^{2})}}\right)}{2\pi (1-\rho_{z}^{2})}. \end{array}$$

Now, we consider the expected distortion. First, choose the reconstruction function

(79) $$\begin{array}{c} {\hat{\tilde{X}}}_{1}\left(U,Y\right)=U \end{array}$$

for the binary uniformly distributed *U* and the channel $P_{\tilde{X}|U}$ such that $\mathrm{Pr}[\tilde{X}\neq U]=q$ for any q∈[0,0.5], as considered above. For this reconstruction function and choices of *U* and $P_{\tilde{X}|U}$, we obtain the expected distortion

(80) $$\begin{array}{c} E\left[d\left(\tilde{X},{\hat{\tilde{X}}}_{1}\left(U,Y\right)\right)\right]=q. \end{array}$$

Second, choose the reconstruction function

(81) $$\begin{array}{c} {\hat{\tilde{X}}}_{2}\left(U,Y\right)=\mathrm{sgn}\left(Y\right) \end{array}$$

and consider *U*. We then obtain

(82) $$\begin{array}{c} E\left[d\left(\tilde{X},{\hat{\tilde{X}}}_{2}\left(U,Y\right)\right)\right]=p*Q\left(\frac{\rho_{y}}{\sqrt{1-\rho_{y}^{2}}}\right) \end{array}$$

which follows since the channel $P_{\mathrm{sgn}\left(Y\right)|\tilde{X}}$ can be considered as a concatenation of two BSCs with crossover probabilities *p* and $Q\left(\frac{\rho_{y}}{\sqrt{1-\rho_{y}^{2}}}\right)$, where the former follows since $\mathrm{Pr}[\tilde{X}\neq X]=p$ and the latter because X∈{−1,1} and

(83) $$\begin{array}{c} \mathrm{Pr}\left[X\neq \mathrm{sgn}\left(Y\right)\right]=\mathrm{Pr}\left[X\neq \mathrm{sgn}\left(\rho_{y}X+N_{y}\right)\right]=\mathrm{Pr}\left[N_{y}>\rho_{y}\right]. \end{array}$$

Therefore, the proof for the achievable lossy secure and private source coding region follows by combining (68)–(70), (72), (74), (77), (80), and (82) by applying time sharing, with time-sharing parameter λ∈[0,1], between the two reconstruction functions in (79) and (81) with corresponding *U* and $P_{\tilde{X}|U}$, since for constant *U* the terms in (25)–(27) are zero. □

**Remark** **1.** *The proof of Proposition 3 follows similar steps as those in [46] (Section II) and it seems that the achievable lossy secure and private source coding region given in Proposition 3 is optimal. Considering $\left(R_{w},R_{s},R_{\ell }\right)$, one can apply Mrs. Gerber’s lemma to show that the choice of U such that $P_{\tilde{X}|U}$ is a BSC(q) after relabeling the input and output symbols is optimal, since Mrs. Gerber’s lemma is valid for all binary-input symmetric memoryless channels with discrete or continuous outputs [47]. This result follows because convexity is preserved; see also [48] (Appendix B) for an alternative proof of convexity preservation for independent BSC measurements. However, it is not entirely clear how to prove that the sign operation used for estimation suffices for the rate region.*

## 6. Proof for Theorem 1

### 6.1. Achievability Proof for Theorem 1

**Proof** **Sketch.** We leverage the output statistics of random binning (OSRB) method [16,49,50] for the achievability proof by following the steps described in [51] (Section 1.6).

Let $\left(V^{n},U^{n},{\tilde{X}}^{n},X^{n},Y^{n},Z^{n}\right)$ be i.i.d. according to $P_{VU\tilde{X}XYZ}$ that can be obtained from (14) by fixing $P_{U|\tilde{X}}$ and $P_{V|U}$, such that $E\left[d(\tilde{X},\hat{\tilde{X}}\right)]\leq \left(D+\epsilon \right)$ for any ϵ>0. To each $v^{n}$ assign two random bin indices $F_{v}\in \left[1:2^{n{\tilde{R}}_{v}}\right]$ and $W_{v}\in \left[1:2^{nR_{v}}\right]$. Furthermore, to each $u^{n}$ assign three random bin indices $F_{u}\in \left[1:2^{n{\tilde{R}}_{u}}\right]$, $W_{u}\in \left[1:2^{nR_{u}}\right]$, and $K_{u}\in \left[1:\;2^{nR_{0}}\right]$, where $R_{0}$ is the private key rate defined in Section 2. Public indices $F=(F_{v},F_{u})$ represent the choice of a source encoder and decoder pair. Furthermore, we impose that the messages sent by the source encoder Enc(·,·) to the source decoder Dec(·,·,·) are

(84) $$\begin{array}{cc} \; & W=(W_{v},W_{u},K+K_{u}) \end{array}$$

where the summation with the private key is in modulo- $2^{nR_{0}}$, i.e., one-time padding.

The public index $F_{v}$ is almost independent of $\left({\tilde{X}}^{n},X^{n},Y^{n},Z^{n}\right)$ if we have [49] (Theorem 1)

(85) $$\begin{array}{c} {\tilde{R}}_{v}<H\left(V|\tilde{X},X,Y,Z\right)\overset{\left(a\right)}{=}H\left(V|\tilde{X}\right) \end{array}$$

where (a) follows since $\left(X,Y,Z\right)-\tilde{X}-V$ form a Markov chain. The constraint in (85) suggests that the expected value, taken over the random bin assignments, of the variational distance between the joint probability distributions $\mathrm{Unif}\left[1\;\;:\;2^{n{\tilde{R}}_{v}}\right]\cdot \;P_{{\tilde{X}}^{n}}$ and $P_{F_{v}{\tilde{X}}^{n}}$ vanishes when n→∞. Moreover, the public index $F_{u}$ is almost independent of $\left(V^{n},{\tilde{X}}^{n},X^{n},Y^{n},Z^{n}\right)$ if we have

(86) $$\begin{array}{c} {\tilde{R}}_{u}<H\left(U|V,\tilde{X},X,Y,Z\right)\overset{\left(a\right)}{=}H\left(U|V,\tilde{X}\right) \end{array}$$

where (a) follows from the Markov chain relation $\left(X,Y,Z\right)-\tilde{X}-\left(U,V\right)$.

Using a Slepian–Wolf (SW) [1] decoder that observes $\left(Y^{n},F_{v},W_{v}\right)$, one can reliably estimate $V^{n}$ if we have [49] (Lemma 1)

(87) $$\begin{array}{c} {\tilde{R}}_{v}+R_{v}>H\left(V|Y\right) \end{array}$$

since then the expected error probability, taken over random bin assignments, vanishes when n→∞. Furthermore, one can reliably estimate $U^{n}$ by using a SW decoder that observes $\left(K,V^{n},Y^{n},F_{u},W_{u},K+K_{u}\right)$ if we have

(88) $$\begin{array}{cc} \; & R_{0}+{\tilde{R}}_{u}+R_{u}>H\left(U|V,Y\right). \end{array}$$

To satisfy (85)–(88), for any ϵ>0 we fix

(89) $$\begin{array}{cc} \; & {\tilde{R}}_{v}=H\left(V|\tilde{X}\right)-\epsilon \end{array}$$

(90) $$\begin{array}{cc} \; & R_{v}=I\left(V;\tilde{X}\right)-I\left(V;Y\right)+2\epsilon \end{array}$$

(91) $$\begin{array}{cc} \; & {\tilde{R}}_{u}=H\left(U|V,\tilde{X}\right)-\epsilon \end{array}$$

(92) $$\begin{array}{cc} \; & R_{0}+R_{u}=I\left(U;\tilde{X}|V\right)-I\left(U;Y|V\right)+2\epsilon . \end{array}$$

Since all tuples $\left(v^{n},u^{n},{\tilde{x}}^{n},x^{n},y^{n},z^{n}\right)$ are in the jointly typical set with high probability, by the typical average lemma [2] (p. 26), the distortion constraint (4) is satisfied.

**Communication Rate**: (90) and (92) result in a communication (storage) rate of

(93) $$\begin{array}{cc} \; & R_{w}=R_{0}+R_{v}+R_{u}\overset{\left(a\right)}{=}I\left(U;\tilde{X}|Y\right)+4\epsilon \end{array}$$

where (a) follows since $V-U-\tilde{X}-Y$ form a Markov chain.

**Privacy Leakage Rate**: Since private key *K* is uniformly distributed, and is independent of source and channel random variables, we can consider the following virtual scenario to calculate the leakage. We first assume for the virtual scenario that there is no private key such that the encoder output for the virtual scenario is

(94) $$\begin{array}{cc} \; & \bar{W}=(W_{v},W_{u},K_{u}). \end{array}$$

We calculate the leakage for the virtual scenario. Then, given the mentioned properties of the private key and due to the one-time padding step in (84), we can subtract $H\left(K\right)=nR_{0}$ from the leakage calculated for the virtual scenario to obtain the leakage for the original problem, which follows from the sum of (91) and (92) if ϵ→0 when n→∞. Thus, we have the privacy leakage

(95) $$\begin{array}{cc} \; & I\left(X^{n};W,F|Z^{n}\right)=I\left(X^{n};\bar{W},F|Z^{n}\right)-nR_{0}\\ \; & \overset{\left(a\right)}{=}\;H\left(\bar{W},F|Z^{n}\right)\;-\;H\left(\bar{W},F|X^{n}\right)\;-\;nR_{0}\\ \; & \overset{\left(b\right)}{=}H\left(\bar{W},F|Z^{n}\right)-H\left(U^{n},V^{n}|X^{n}\right)+H\left(V^{n}|\bar{W},F,X^{n}\right)+H\left(U^{n}|V^{n},\bar{W},F,X^{n}\right)-nR_{0}\\ \; & \overset{\left(c\right)}{\leq }H\left(\bar{W},F|Z^{n}\right)-nH\left(U,V|X\right)+2n\epsilon_{n}-nR_{0} \end{array}$$

where (a) follows because $\left(\bar{W},F\right)-X^{n}-Z^{n}$ form a Markov chain, (b) follows since $\left(U^{n},V^{n}\right)$ determine $\left(F_{u},W_{u},K_{u},F_{v},W_{v}\right)$, and (c) follows since $\left(U^{n},V^{n},X^{n}\right)$ is i.i.d. and for some $\epsilon_{n}>0$ such that $\epsilon_{n}\to 0$ when n→∞ because $\left(F_{v},W_{v},X^{n}\right)$ can reliably recover $V^{n}$ by (87) because of the Markov chain relation $V^{n}-X^{n}-Y^{n}$ and, similarly, $\left(F_{u},W_{u},K_{u},V^{n},X^{n}\right)$ can reliably recover $U^{n}$ by (88) because of H(U|V,Y)≥H(U|V,X) that is proved in [21] (Equation (55)) for the Markov chain relation (V,U)−X−Y.

Next, we consider the term $H(\bar{W},F|Z^{n})$ in (95) and provide single letter bounds on it by applying the six different decodability results given in [21] (Section V-A) that are applied to an entirely similar conditional entropy term in [21] (Equation (54)) that measures the uncertainty in indices conditioned on an i.i.d. multi-letter random variable. Thus, combining the six decodability results in [21] (Section V-A) with (95) we obtain

(96) $$\begin{array}{cc} \; & I\left(X^{n};W,F|Z^{n}\right)\leq n\left({\left[I\left(U;Z|V\right)-I\left(U;Y|V\right)+\epsilon \right]}^{-}+I\left(U;X|Z\right)+3\epsilon_{n}-R_{0}\right). \end{array}$$

The equation (92) implicitly assumes that private key rate $R_{0}$ is less than $\left(I\left(U;\tilde{X}|V\right)\;-\;I\left(U;Y|V\right)\;+\;\;2\epsilon \right)$$=(I\left(U;\tilde{X}|Y,V\right)+2\epsilon )$, where the equality follows from the Markov chain relation $\left(V,U\right)-\tilde{X}-Y$. The communication rate results are not affected by this assumption, since ${\tilde{X}}^{n}$ should be reconstructed by the decoder. However, if the private key rate $R_{0}$ is greater than or equal to $\left(I\left(U;\tilde{X}|Y,V\right)+2\epsilon \right)$, then we can remove the bin index $K_{u}$ from the code construction above and apply one-time padding to the bin index $W_{u}$, such that we have the encoder output

(97) $$\begin{array}{cc} \; & \overset{=}{W}=(W_{v},W_{u}+K) \end{array}$$

where the summation with the private key is in modulo- $2^{nR_{u}}=2^{n(I\left(U;\tilde{X}|Y,V\right)+2\epsilon )}$. Thus, one then does not leak any information about $W_{u}$ to the eavesdropper because of the one-time padding step in (97). We then have privacy leakage

(98) $$\begin{array}{cc} \; & I\left(X^{n};\overset{=}{W},F|Z^{n}\right)=I\left(X^{n};W_{v},F|Z^{n}\right)\\ \; & \overset{\left(a\right)}{\leq }H\left(X^{n}|Z^{n}\right)-H\left(X^{n}|Z^{n},W_{v},F_{v}\right)+\epsilon_{n}^{\prime }\\ \; & \overset{\left(b\right)}{\leq }H\left(X^{n}|Z^{n}\right)-H\left(X^{n}|Z^{n},V^{n}\right)+\epsilon_{n}^{\prime }\\ \; & \overset{\left(c\right)}{=}nI\left(V;X|Z\right)+\epsilon_{n}^{\prime } \end{array}$$

where (a) follows for some $\epsilon_{n}^{\prime }$ such that $\epsilon_{n}^{\prime }\to 0$ when n→∞ since by (86) $F_{u}$ is almost independent of $\left(V^{n},X^{n},Z^{n}\right)$; see also [52] (Theorem 1), (b) follows since $V^{n}$ determines $\left(F_{v},W_{v}\right)$, and (c) follows because $\left(X^{n},Z^{n},V^{n}\right)$ are i.i.d.

Note we can reduce the privacy leakage given in (98) if $R_{0}\geq \left(I\left(U;\tilde{X}\right)-I\left(U;Y\right)+4\epsilon \right)=\left(I\left(U;\tilde{X}|Y\right)+4\epsilon \right)$, where the equality follows from the Markov chain relation $U-\tilde{X}-Y$, since then we can apply one-time padding to both bin indices $W_{v}$ and $W_{u}$ with the sum rate

(99) $$\begin{array}{cc} \; & R_{v}+R_{u}\overset{\left(a\right)}{=}I\left(V;\tilde{X}\right)-I\left(V;Y\right)+2\epsilon \;+\;I\left(U;\tilde{X}|V\right)\;-\;I\left(U;Y|V\right)\;+\;2\epsilon \\ \; & \overset{\left(b\right)}{=}I\left(U;\tilde{X}\right)-I\left(U;Y\right)+4\epsilon \end{array}$$

where (a) follows by (90) and (92), and (b) follows from the Markov chain relation $V-U-\tilde{X}-Y$. Thus, one then does not leak any information about $\left(W_{v},W_{u}\right)$ to the eavesdropper because of the one-time padding step, so we then obtain the privacy leakage of

(100) $$\begin{array}{cc} \; & I\left(X^{n};F|Z^{n}\right)=I\left(X^{n};F_{v}|Z^{n}\right)+I\left(X^{n};F_{u}|Z^{n},F_{v}\right)\overset{\left(a\right)}{\leq }2\epsilon_{n}^{\prime } \end{array}$$

where (a) follows since by (85) $F_{v}$ is almost independent of $\left(X^{n},Z^{n}\right)$ and by (86) $F_{u}$ is almost independent of $\left(V^{n},X^{n},Z^{n}\right)$.

**Secrecy Leakage Rate**: Similar to the privacy leakage analysis above, we first consider the virtual scenario with the encoder output given in (94), and then calculate the leakage for the original problem by subtracting $H\left(K\right)=nR_{0}$ from the leakage calculated for the virtual scenario. Thus, we obtain

(101) $$\begin{array}{cc} \; & I\left({\tilde{X}}^{n};W,F|Z^{n}\right)=I\left({\tilde{X}}^{n};\bar{W},F|Z^{n}\right)-nR_{0}\\ \; & \overset{\left(a\right)}{=}H\left(\bar{W},F|Z^{n}\right)-H\left(\bar{W},F|{\tilde{X}}^{n}\right)-nR_{0}\\ \; & \overset{\left(b\right)}{=}H\left(\bar{W},F|Z^{n}\right)-H\left(U^{n},V^{n}|{\tilde{X}}^{n}\right)+H\left(V^{n}|\bar{W},F,{\tilde{X}}^{n}\right)+H\left(U^{n}|V^{n},\bar{W},F,{\tilde{X}}^{n}\right)\\ \; & \overset{\left(c\right)}{\leq }H\left(\bar{W},F|Z^{n}\right)-nH\left(U,V|\tilde{X}\right)+2n\epsilon_{n}^{\prime }-nR_{0}\\ \; & \overset{\left(d\right)}{\leq }n\left({\left[I\left(U;Z|V\right)-I\left(U;Y|V\right)+\epsilon \right]}^{-}+I\left(U;\tilde{X}|Z\right)+3\epsilon_{n}^{\prime }-R_{0}\right) \end{array}$$

where (a) follows from the Markov chain relation $\left(\bar{W},F\right)-{\tilde{X}}^{n}-Z^{n}$, (b) follows since $\left(U^{n},V^{n}\right)$ determine $\left(\bar{W},F\right)$, (c) follows because $\left(V^{n},U^{n},{\tilde{X}}^{n}\right)$ are i.i.d. and because $\left(F_{v},W_{v},{\tilde{X}}^{n}\right)$ can reliably recover $V^{n}$ by (87) due to the Markov chain relation $V^{n}-{\tilde{X}}^{n}-Y^{n}$ and, similarly, $\left(F_{u},W_{u},K_{u},V^{n},{\tilde{X}}^{n}\right)$ can reliably recover $U^{n}$ by (88) due to $H\left(U|V,Y\right)\;\geq \;H\left(U|V,\tilde{X}\right)$ that can be proved as in [21] (Equation (55)) for the Markov chain relation $\left(V,U\right)-\tilde{X}-Y$, and (d) follows by applying the six decodability results in [21] (Section V-A) that are applied to (95) with the final result in (96) by replacing *X* with $\tilde{X}$.

Similar to the privacy leakage analysis above, if we have $R_{0}\geq \left(I\left(U;\tilde{X}|Y,V\right)+2\epsilon \right)$, then we can eliminate $K_{u}$ and apply one-time padding as in (97), such that no information about $W_{u}$ is leaked to the eavesdropper, we have

(102) $$\begin{array}{cc} \; & I\left({\tilde{X}}^{n};\overset{=}{W},F|Z^{n}\right)=I\left({\tilde{X}}^{n};W_{v},F|Z^{n}\right)\\ \; & \overset{\left(a\right)}{\leq }H\left({\tilde{X}}^{n}|Z^{n}\right)-H\left({\tilde{X}}^{n}|Z^{n},W_{v},F_{v}\right)+\epsilon_{n}^{\prime }\\ \; & \overset{\left(b\right)}{\leq }H\left({\tilde{X}}^{n}|Z^{n}\right)-H\left({\tilde{X}}^{n}|Z^{n},V^{n}\right)+\epsilon_{n}^{\prime }\\ \; & \overset{\left(c\right)}{=}nI\left(V;\tilde{X}|Z\right)+\epsilon_{n}^{\prime } \end{array}$$

where (a) follows because by (86) $F_{u}$ is almost independent of $\left(V^{n},{\tilde{X}}^{n},Z^{n}\right)$, (b) follows since $V^{n}$ determines $\left(F_{v},W_{v}\right)$, and (c) follows because $\left({\tilde{X}}^{n},Z^{n},V^{n}\right)$ are i.i.d.

If $R_{0}\geq \left(I\left(U;\tilde{X}|Y\right)+4\epsilon \right)$, we can apply one-time padding to hide $\left(W_{v},W_{u}\right)$, as in the privacy leakage analysis above. We then have the secrecy leakage of

(103) $$\begin{array}{cc} \; & I\left({\tilde{X}}^{n};F|Z^{n}\right)=I\left({\tilde{X}}^{n};F_{v}|Z^{n}\right)+I\left({\tilde{X}}^{n};F_{u}|Z^{n},F_{v}\right)\overset{\left(a\right)}{\leq }2\epsilon_{n}^{\prime } \end{array}$$

where (a) follows since by (85) $F_{v}$ is almost independent of $\left({\tilde{X}}^{n},Z^{n}\right)$ and by (86) $F_{u}$ is almost independent of $\left(V^{n},{\tilde{X}}^{n},Z^{n}\right)$.

Suppose that public indices *F* are generated uniformly at random, and the encoder generates $\left(V^{n},U^{n}\right)$ according to $P_{V^{n}U^{n}|{\tilde{X}}^{n}F_{v}F_{u}}$ that can be obtained from the proposed binning scheme above to compute the bins $W_{v}$ from $V^{n}$ and $W_{u}$ from $U^{n}$, respectively. Such a procedure results in a joint probability distribution almost equal to $P_{VU\tilde{X}XYZ}$ fixed above [51] (Section 1.6). The privacy and secrecy leakage metrics above are expectations over all possible public index realizations F=f. Therefore, using a time-sharing random variable *Q* for convexification and applying the selection lemma [53] (Lemma 2.2) to each decodability case separately, the achievability for Theorem 1 follows by choosing an ϵ>0 such that ϵ→0 when n→∞. □

### 6.2. Converse Proof for Theorem 1

**Proof** **Sketch.** Assume that for some $\delta_{n}\;>\;0$ and n≥1, there exist an encoder and a decoder, such that (1)–(4) are satisfied for some tuple $\left(R_{w},R_{s},R_{\ell },D\right)$ given a private key with rate $R_{0}$.

Define $V_{i}≜\left(W,Y_{i+1}^{n},Z^{i-1}\right)$ and $U_{i}≜\left(W,Y_{i+1}^{n},Z^{i-1},X^{i-1},K\right)$ that satisfy the Markov chain relation $V_{i}-U_{i}-{\tilde{X}}_{i}-X_{i}-\left(Y_{i},Z_{i}\right)$ by definition of the source statistics. We have

(104) $$\begin{array}{cc} \; & D+\delta_{n}\overset{\left(a\right)}{\geq }E\left[d\left({\tilde{X}}^{n},\hat{{\tilde{X}}^{n}}\left(Y^{n},W,K\right)\right)\right]\\ \; & \overset{\left(b\right)}{\geq }E\left[d\left({\tilde{X}}^{n},\hat{{\tilde{X}}^{n}}\left(Y^{n},W,K,X^{i-1},Z^{i-1}\right)\right)\right]\\ \; & \overset{\left(c\right)}{=}E\left[d\left({\tilde{X}}^{n},\hat{{\tilde{X}}^{n}}\left(Y_{i}^{n},W,K,X^{i-1},Z^{i-1}\right)\right)\right]\\ \; & \overset{\left(d\right)}{=}\frac{1}{n}\sum_{i=1}^{n}E\left[d\left({\tilde{X}}_{i},\hat{{\tilde{X}}_{i}}\left(U_{i},Y_{i}\right)\right)\right] \end{array}$$

where (a) follows by (4), (b) follows since providing more information to the reconstruction function does not increase expected distortion, (c) follows from the Markov chain relation

(105) $$\begin{array}{c} Y^{i-1}-\left(Y_{i}^{n},X^{i-1},Z^{i-1},W,K\right)-{\tilde{X}}^{n} \end{array}$$

and (d) follows from the definition of $U_{i}$.

**Communication Rate**: For any $R_{0}\geq 0$, we have

$$\begin{array}{cc} \; & n\left(R_{w}+\delta_{n}\right)\overset{\left(a\right)}{\geq }\log\left|W\right| \end{array}$$

(106) $$\begin{array}{cc} \; & \geq H\left(W|Y^{n},K\right)-H\left(W|Y^{n},K,{\tilde{X}}^{n}\right) \end{array}$$

(107) $$\begin{array}{cc} \; & \overset{\left(b\right)}{=}\sum_{i=1}^{n}I\left(W;{\tilde{X}}_{i}|{\tilde{X}}^{i-1},Y_{i+1}^{n},Z^{i-1},K,Y_{i}\right)\\ \; & \overset{\left(c\right)}{=}\sum_{i=1}^{n}I\left({\tilde{X}}^{i-1},Y_{i+1}^{n},Z^{i-1},K,W;{\tilde{X}}_{i}|Y_{i}\right)\\ \; & \overset{\left(d\right)}{\geq }\sum_{i=1}^{n}I\left(X^{i-1},Y_{i+1}^{n},Z^{i-1},K,W;{\tilde{X}}_{i}|Y_{i}\right) \end{array}$$

(108) $$\begin{array}{cc} \; & \overset{\left(e\right)}{=}\sum_{i=1}^{n}I\left(U_{i};{\tilde{X}}_{i}|Y_{i}\right) \end{array}$$

where (a) follows by (1), (b) follows from the Markov chain relation

(109) $$\begin{array}{c} \left(Y^{i-1},X^{i-1},Z^{i-1}\right)-\left({\tilde{X}}^{i-1},Y_{i}^{n},K\right)-\left({\tilde{X}}_{i},W\right) \end{array}$$

(c) follows because $\left({\tilde{X}}_{i},Y_{i}\right)$ are independent of $\left({\tilde{X}}^{i-1},Y_{i+1}^{n},Z^{i-1},K\right)$, (d) follows by applying the data processing inequality to the Markov chain relation in (109), and (e) follows from the definition of $U_{i}$.

**Privacy Leakage Rate**: We obtain

$$\begin{array}{cc} \; & n(R_{\ell }+\delta_{n})\\ \; & \overset{\left(a\right)}{\geq }\left[I\left(W;Y^{n}\right)-I\left(W;Z^{n}\right)\right]+\left[I\left(W;X^{n}\right)-I\left(W;Y^{n}\right)\right]\\ \; & \overset{\left(b\right)}{=}\left[I\left(W;Y^{n}\right)-I\left(W;Z^{n}\right)\right]+I\left(W;X^{n}|K\right)-I\left(K;X^{n}|W\right)-I\left(W;Y^{n}|K\right)+I\left(K;Y^{n}|W\right)\\ \; & \overset{\left(c\right)}{=}\left[I\left(W;Y^{n}\right)-I\left(W;Z^{n}\right)\right]+\left[I\left(W;X^{n}|K\right)-I\left(W;Y^{n}|K\right)\right]-I\left(K;X^{n}|W,Y^{n}\right)\\ \; & \geq \sum_{i=1}^{n}\left[I\left(W;Y_{i}|Y_{i+1}^{n}\right)-I\left(W;Z_{i}|Z^{i-1}\right)\right]\\ \; & \;+\sum_{i=1}^{n}\left[I\left(W;X_{i}|X^{i-1},K\right)-I\left(W;Y_{i}|Y_{i+1}^{n},K\right)\right]-H\left(K\right)\\ \; & \overset{\left(d\right)}{=}\sum_{i=1}^{n}\left[I\left(W;Y_{i}|Y_{i+1}^{n},Z^{i-1}\right)-I\left(W;Z_{i}|Z^{i-1},Y_{i+1}^{n}\right)-R_{0}\right]\\ \; & \;+\sum_{i=1}^{n}\left[I\left(W;X_{i}|X^{i-1},Y_{i+1}^{n},K\right)-I\left(W;Y_{i}|Y_{i+1}^{n},X^{i-1},K\right)\right]\\ \; & \overset{\left(e\right)}{=}\;\sum_{i=1}^{n}\left[I\left(W;Y_{i}|Y_{i+1}^{n},Z^{i-1}\right)\;-\;I\left(W;Z_{i}|Z^{i-1},Y_{i+1}^{n}\right)\;-\;R_{0}\right]\\ \; & \;+\sum_{i=1}^{n}\left[I\left(W;X_{i}|X^{i-1},Y_{i+1}^{n},Z^{i-1},K\right)-I\left(W;Y_{i}|Y_{i+1}^{n},X^{i-1},Z^{i-1},K\right)\right]\\ \; & \overset{\left(f\right)}{=}\sum_{i=1}^{n}\left[I\left(W,Y_{i+1}^{n},Z^{i-1};Y_{i}\right)\;-\;I\left(W,Z^{i-1},Y_{i+1}^{n};Z_{i}\right)-R_{0}\right]\\ \; & \;+\sum_{i=1}^{n}\left[I\left(W,X^{i-1},Y_{i+1}^{n},Z^{i-1},K;X_{i}\right)-I\left(W,Y_{i+1}^{n},X^{i-1},Z^{i-1},K;Y_{i}\right)\right] \end{array}$$

(110) $$\begin{array}{cc} \; & \overset{\left(g\right)}{=}\sum_{i=1}^{n}\left[I\left(V_{i};Y_{i}\right)-I\left(V_{i};Z_{i}\right)-R_{0}+I\left(U_{i},V_{i};X_{i}\right)-I\left(U_{i},V_{i};Y_{i}\right)\right]\\ \; & =\sum_{i=1}^{n}\left[-I\left(U_{i},V_{i};Z_{i}\right)-R_{0}+I\left(U_{i},V_{i};X_{i}\right)+\left(I\left(U_{i};Z_{i}|V_{i}\right)-I\left(U_{i};Y_{i}|V_{i}\right)\right)\right]\\ \; & \overset{\left(h\right)}{\geq }\;\sum_{i=1}^{n}\left[I\left(U_{i};X_{i}|Z_{i}\right)-R_{0}+{\left[I\left(U_{i};Z_{i}|V_{i}\right)-I\left(U_{i};Y_{i}|V_{i}\right)\right]}^{-}\right] \end{array}$$

where (a) follows by (3) and from the Markov chain relation $W-X^{n}-Z^{n}$, (b) follows since *K* is independent of $\left(X^{n},Y^{n}\right)$, (c) follows from the Markov chain relation $\left(W,K\right)-X^{n}-Y^{n}$, (d) follows because $H\left(K\right)=nR_{0}$ and from Csiszár’s sum identity [54], (e) follows from the Markov chain relations

(111) $$\begin{array}{cc} \; & Z^{i-1}-\left(X^{i-1},Y_{i+1}^{n},K\right)-\left(X_{i},W\right) \end{array}$$

(112) $$\begin{array}{cc} \; & Z^{i-1}-\left(X^{i-1},Y_{i+1}^{n},K\right)-\left(Y_{i},W\right) \end{array}$$

(f) follows because $\left(X^{n},Y^{n},Z^{n}\right)$ are i.i.d. and *K* is independent of $\left(X^{n},Y^{n},Z^{n}\right)$, (g) follows from the definitions of $V_{i}$ and $U_{i}$, and (h) follows from the Markov chain relation $V_{i}-U_{i}-X_{i}-Z_{i}$.

Next, we provide the matching converse for the privacy leakage rate in (98), which is achieved when $R_{0}\geq I\left(U;\tilde{X}|Y,V\right)$. We have

(113) $$\begin{array}{cc} \; & n\left(R_{\ell }+\delta_{n}\right)\overset{\left(a\right)}{\geq }H\left(X^{n}|Z^{n}\right)-H\left(X^{n}|Z^{n},W\right)\\ \; & \overset{\left(b\right)}{=}H\left(X^{n}|Z^{n}\right)-\sum_{i=1}^{n}H\left(X_{i}|Z_{i},Z^{i-1},X_{i+1}^{n},W,Y_{i+1}^{n}\right)\\ \; & \overset{\left(c\right)}{=}H\left(X^{n}|Z^{n}\right)-\sum_{i=1}^{n}H\left(X_{i}|Z_{i},V_{i},X_{i+1}^{n}\right)\\ \; & \overset{\left(d\right)}{\geq }\sum_{i=1}^{n}\left[H\left(X_{i}|Z_{i}\right)-H\left(X_{i}|Z_{i},V_{i}\right)\right]\\ \; & =\sum_{i=1}^{n}I\left(V_{i};X_{i}|Z_{i}\right) \end{array}$$

where (a) follows by (3), (b) follows from the Markov chain relation

(114) $$\begin{array}{c} \left(Z_{i+1}^{n},Y_{i+1}^{n}\right)-\left(X_{i+1}^{n},W,Z^{i}\right)-X_{i} \end{array}$$

(c) follows from the definition of $V_{i}$, and (d) follows because $\left(X^{n},Z^{n}\right)$ are i.i.d.

The matching converse for the privacy leakage rate in (100), achieved when $R_{0}\geq I\left(U;\tilde{X}|Y\right)$, follows from the fact that conditional mutual information is non-negative.

**Secrecy Leakage Rate**: We have

$$\begin{array}{cc} \; & n(R_{s}+\delta_{n})\\ \; & \overset{\left(a\right)}{\geq }\left[I\left(W;Y^{n}\right)-I\left(W;Z^{n}\right)\right]+\left[I\left(W;{\tilde{X}}^{n}\right)-I\left(W;Y^{n}\right)\right]\\ \; & \overset{\left(b\right)}{=}\left[I\left(W;Y^{n}\right)-I\left(W;Z^{n}\right)\right]+I\left(W;{\tilde{X}}^{n}|K\right)-I\left(K;{\tilde{X}}^{n}|W\right)-I\left(W;Y^{n}|K\right)+I\left(K;Y^{n}|W\right)\\ \; & \overset{\left(c\right)}{=}\left[I\left(W;Y^{n}\right)-I\left(W;Z^{n}\right)\right]+\left[I\left(W;{\tilde{X}}^{n}|K\right)-I\left(W;Y^{n}|K\right)\right]-I\left(K;{\tilde{X}}^{n}|W,Y^{n}\right)\\ \; & \overset{\left(d\right)}{\geq }\sum_{i=1}^{n}\left[I\left(W;Y_{i}|Y_{i+1}^{n}\right)-I\left(W;Z_{i}|Z^{i-1}\right)\right]+I\left(W;{\tilde{X}}^{n}|Y^{n},K\right)-\;H\left(K\right) \end{array}$$

(115) $$\begin{array}{cc} \; & \overset{\left(e\right)}{=}\sum_{i=1}^{n}\left[I\left(W;Y_{i}|Y_{i+1}^{n},Z^{i-1}\right)-I\left(W;Z_{i}|Z^{i-1},Y_{i+1}^{n}\right)-R_{0}\right]\\ \; & \;+nH\left(\tilde{X}|Y\right)-\sum_{i=1}^{n}H\left({\tilde{X}}_{i}|Y_{i},Y_{i+1}^{n},W,K,{\tilde{X}}^{i-1}\right)\\ \; & \overset{\left(f\right)}{\geq }\sum_{i=1}^{n}\left[I\left(W,Y_{i+1}^{n},Z^{i-1};Y_{i}\right)-I\left(W,Z^{i-1},Y_{i+1}^{n};Z_{i}\right)-R_{0}\right]\\ \; & \;+nH\left(\tilde{X}|Y\right)-\sum_{i=1}^{n}H\left({\tilde{X}}_{i}|Y_{i},Y_{i+1}^{n},W,K,X^{i-1},Z^{i-1}\right)\\ \; & \overset{\left(g\right)}{=}\sum_{i=1}^{n}\left[I\left(V_{i};Y_{i}\right)-I\left(V_{i};Z_{i}\right)-R_{0}\right]+nH\left(\tilde{X}|Y\right)-\sum_{i=1}^{n}H\left({\tilde{X}}_{i}|Y_{i},U_{i},V_{i}\right)\\ \; & \overset{\left(h\right)}{=}\sum_{i=1}^{n}\left[I\left(V_{i};Y_{i}\right)-I\left(V_{i};Z_{i}\right)-R_{0}\right]+\sum_{i=1}^{n}\left[I\left(U_{i},V_{i};{\tilde{X}}_{i}\right)-I\left(U_{i},V_{i};Y_{i}\right)\right]\\ \; & =\sum_{i=1}^{n}\left[-I\left(U_{i},V_{i};Z_{i}\right)-R_{0}+I\left(U_{i},V_{i};{\tilde{X}}_{i}\right)+\left(I\left(U_{i};Z_{i}|V_{i}\right)-I\left(U_{i};Y_{i}|V_{i}\right)\right)\right]\\ \; & \overset{\left(i\right)}{\geq }\sum_{i=1}^{n}\left[I\left(U_{i};{\tilde{X}}_{i}|Z_{i}\right)-R_{0}+{\left[I\left(U_{i};Z_{i}|V_{i}\right)-I\left(U_{i};Y_{i}|V_{i}\right)\right]}^{-}\right] \end{array}$$

where (a) follows by (2) and from the Markov chain relation $W-{\tilde{X}}^{n}-Z^{n}$, (b) follows because *K* is independent of $\left({\tilde{X}}^{n},Y^{n}\right)$, (c) and (d) follow from the Markov chain relation $\left(W,K\right)-{\tilde{X}}^{n}-Y^{n}$, (e) follows because $H\left(K\right)=nR_{0}$ and $\left({\tilde{X}}^{n},Y^{n}\right)$ are i.i.d. and independent of *K*, and from the Csiszár’s sum identity and the Markov chain relation

(116) $$\begin{array}{c} Y^{i-1}-\left({\tilde{X}}^{i-1},W,K,Y_{i+1}^{n},Y_{i}\right)-{\tilde{X}}_{i} \end{array}$$

(f) follows since $\left(Y^{n},Z^{n}\right)$ are i.i.d. and from the data processing inequality applied to the Markov chain relation

(117) $$\begin{array}{c} \left(X^{i-1},Z^{i-1}\right)-\left({\tilde{X}}^{i-1},W,K,Y_{i+1}^{n},Y_{i}\right)-{\tilde{X}}_{i} \end{array}$$

(g) follows from the definitions of $V_{i}$ and $U_{i}$, (h) follows from the Markov chain relation $\left(V_{i},U_{i}\right)-{\tilde{X}}_{i}-Y_{i}$, and (i) follows from the Markov chain relation $V_{i}-U_{i}-{\tilde{X}}_{i}-Z_{i}$.

Next, the matching converse for the secrecy leakage rate in (102), achieved when $R_{0}\geq I\left(U;\tilde{X}|Y,V\right)$, is provided.

(118) $$\begin{array}{cc} \; & n\left(R_{s}+\delta_{n}\right)\overset{\left(a\right)}{\geq }H\left({\tilde{X}}^{n}|Z^{n}\right)-H\left({\tilde{X}}^{n}|Z^{n},W\right)\\ \; & \overset{\left(b\right)}{\geq }H\left({\tilde{X}}^{n}|Z^{n}\right)-\sum_{i=1}^{n}H\left({\tilde{X}}_{i}|Z_{i},Z^{i-1},{\tilde{X}}_{i+1}^{n},W,Y_{i+1}^{n}\right)\\ \; & \overset{\left(c\right)}{=}H\left({\tilde{X}}^{n}|Z^{n}\right)-\sum_{i=1}^{n}H\left({\tilde{X}}_{i}|Z_{i},V_{i},{\tilde{X}}_{i+1}^{n}\right)\\ \; & \overset{\left(d\right)}{\geq }\sum_{i=1}^{n}\left[H\left({\tilde{X}}_{i}|Z_{i}\right)-H\left({\tilde{X}}_{i}|Z_{i},V_{i}\right)\right]=\sum_{i=1}^{n}I\left(V_{i};{\tilde{X}}_{i}|Z_{i}\right) \end{array}$$

where (a) follows by (2), (b) follows from the Markov chain relation

(119) $$\begin{array}{c} \left(Z_{i+1}^{n},Y_{i+1}^{n}\right)-\left({\tilde{X}}_{i+1}^{n},W,Z^{i}\right)-{\tilde{X}}_{i} \end{array}$$

(c) follows from the definition of $V_{i}$, and (d) follows because $\left({\tilde{X}}^{n},Z^{n}\right)$ are i.i.d.

Similar to the privacy leakage analysis above, the matching converse for the secrecy leakage rate in (103), achieved when $R_{0}\geq I\left(U;\tilde{X}|Y\right)$, follows from the fact that conditional mutual information is non-negative. □

Introduce a uniformly distributed time-sharing random variable Q∼Unif[1:n] that is independent of other random variables, and define $X\;=\;X_{Q}$, $\tilde{X}\;=\;{\tilde{X}}_{Q}$, $Y\;=\;Y_{Q}$, $Z\;=\;Z_{Q}$, $V\;=\;V_{Q}$, and $U\;=\;(U_{Q},\;Q)$, so

(120) $$\begin{array}{cc} \; & \left(Q,V\right)\;-U-\tilde{X}-X-\left(Y,Z\right) \end{array}$$

form a Markov chain. The converse proof follows by letting $\delta_{n}\to 0$.

**Cardinality Bounds**: We use the support lemma [54] (Lemma 15.4) for the cardinality bound proofs, which is a standard step, so we omit the proof.

## Data Availability

Not applicable.
